# Supplementary material for: The genetic legacy of legendary and historical Siberian chieftains
Source: Commun Biol. 2020 Oct 16;3:581. doi: 10.1038/s42003-020-01307-3 (PMC7567834; doi:10.1038/s42003-020-01307-3)
Supplement: Supplementary file 1 — Supplementary Information [file 42003_2020_1307_MOESM1_ESM.pdf]

SUPPLEMENTARY INFORMATION

The genetic legacy of legendary and historical Siberian chieftains

Vincent Zvénigorosky*[1,2], Sylvie Duchesne*[3,4], Liubomira Romanova*[3], Patrice Gérard[3], Christiane Petit[5], Michel Petit[5], Anatoly Alexeev[6], Olga Melnichuk[7], Angéla Gonzalez[2], Jean-Luc Fausser[2], Aisen Solovyev[8,9], Georgii Romanov[8,10], Nikolay Barashkov[8,10], Sardana Fedorova[8,10], Bertrand Ludes[1,11], Eric Crubézy[3], Christine Keyser[1,2]

[1] CNRS FRE 2029 BABEL, Paris Descartes University, France

[2] Strasbourg Institute of Legal Medicine, Strasbourg, France

[3] CNRS UMR 5288 AMIS, Toulouse, France

[4] National Institute of Preventive Archaeological Research (INRAP), Cesson-Sévigné, France

[5] French Archaeological Mission in Eastern Siberia (MAFSO)

[6] Institute of Humanities and issues of the minority peoples of the North, Siberian Branch of the Russian Academy of Sciences, Yakutsk, Russian Federation

[7] North-Eastern Federal University. M.K. Ammosova, Yakutsk, Russian Federation

[8] Laboratory of Molecular Biology, Institute of Natural Sciences, MK Ammosov North-Eastern Federal University, Yakutsk, Russia.

[9] Institute for Humanitarian Studies and Problems of Indigenous Peoples of the North, Yakutsk, Russia

[10] Laboratory of Molecular Genetics, Yakut Science Centre of Complex Medical Problems, Yakutsk, Russia

[11] Paris Institute of Legal Medicine, Paris Descartes University, Paris, France

* These authors contributed equally to this work

Corresponding author:

Vincent Zvénigorosky: z.vincent@live.fr

Institut de Médecine Légale, 11 rue Humann, 67000, Strasbourg, France

Supplementary Note 1: Y-haplotype network

The Y-haplotype median-joining network (Supplementary Figure 1) shows that most modern Yakut men carry lineages that were already found in ancient material, especially Ht1. Most other haplotypes fit into the bulk of the Yakut population, around Ht1, Ht2 and Ht3. Notable exceptions include a small branch composed of haplotypes close to those of marginal ancient Yakut men from the pre-1700 phase (Cepzeney and Musee Ethno), which indicates that the presence of these marginal haplotypes (or haplotypes close to them) has been maintained. A second, larger branch comprises a diversity of haplotypes very different from the others. They mostly belong to individuals from the western region (the Vilyuy river basin) and also include three marginal ancient individuals from the pre-1700 phase (Byljasik 3, Oyogosse Tumula 1 and Balyktaek). This branch might be an indicator of the presence of newcomers among modern Yakuts or a strong selection bias in the ancient sample, that led to many ancient men not being buried that would have belonged to that branch, particularly in the western regions. Further archaeological inquiries in the Vilyuy river basin may yield men from these lineages or closely related lineages.

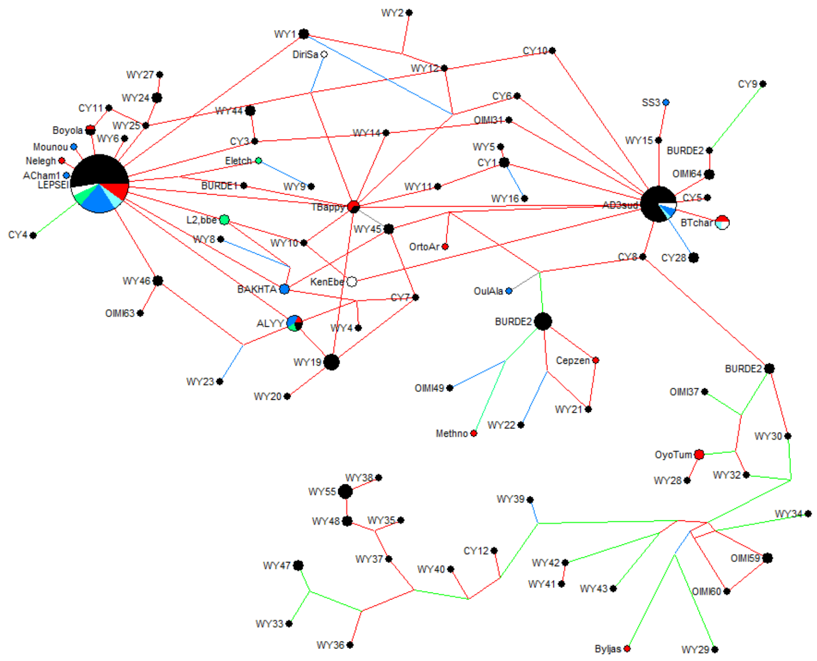

Supplementary Figure 1 – Network of ancient and modern Y-haplotypes

red: anterior to 1700
dark blue: 1700-1750
green: 1750-1800
light blue: 1800-1850
white: 1850-1900
black: posterior to 1900 (modern)
red lines: 1 mutation
blue lines: 2 mutations
green lines: 3 or more mutations

Supplementary Note 2: The At Daban 6 signet rings

Two of the three rings that were deposited in the grave of At Daban 6 could be identified (Supplementary Figure 2 and Supplementary Figure 3). The first was a rendition of the judgment of Solomon and the second a signet ring bearing the arms of the Kvashnin-Samarin, a noble family that held a variety of counsellor or governor positions in the Russian Empire throughout its history.

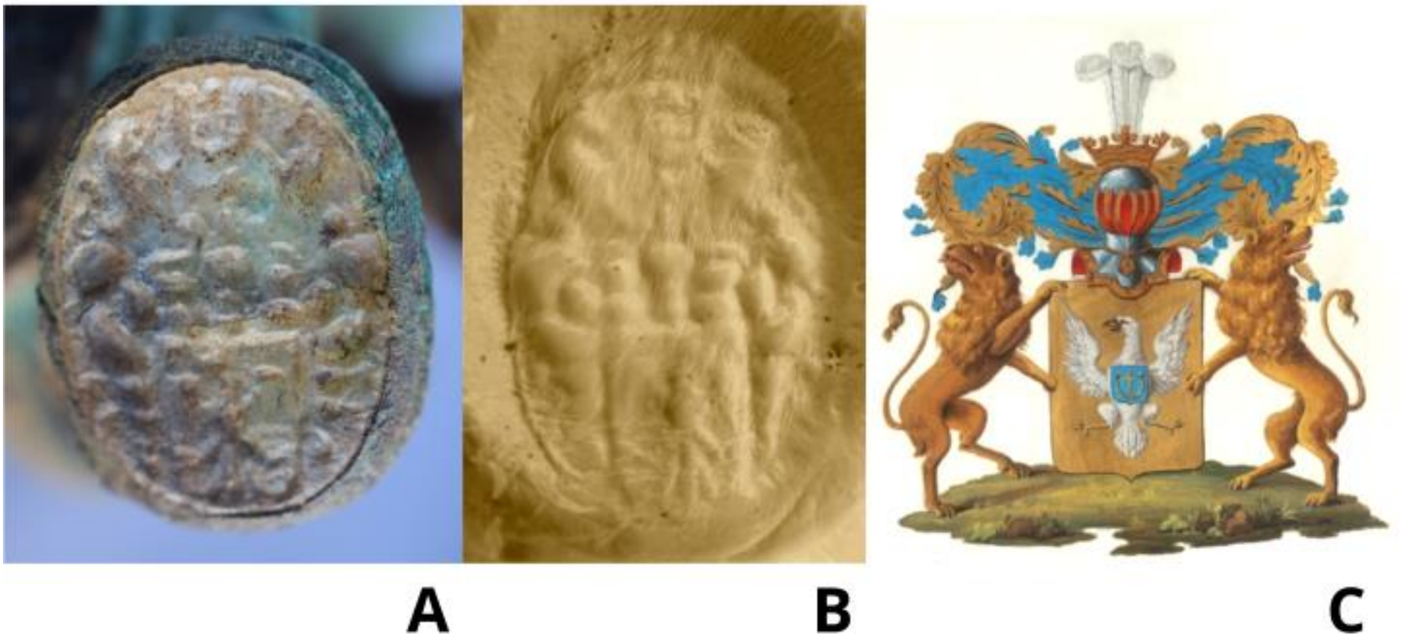

Supplementary Figure 2 – Coat of arms of the Kvashnin-Samarin
A: The embossed signet ring
B: The impression of the engraving, in the manner of a seal
C: A representation of the coat of arms of the Kvashnin-Samarin family (public domain). An escutcheon representing a white single-headed eagle with outstretched wings, supported by two affronted lions rampant, a shield of azure on the eagle's chest bears a golden cross on a silver horseshoe, facing upwards. The crest is a crowned nobleman's helmet, itself with a crest ornate with three ostrich feathers.

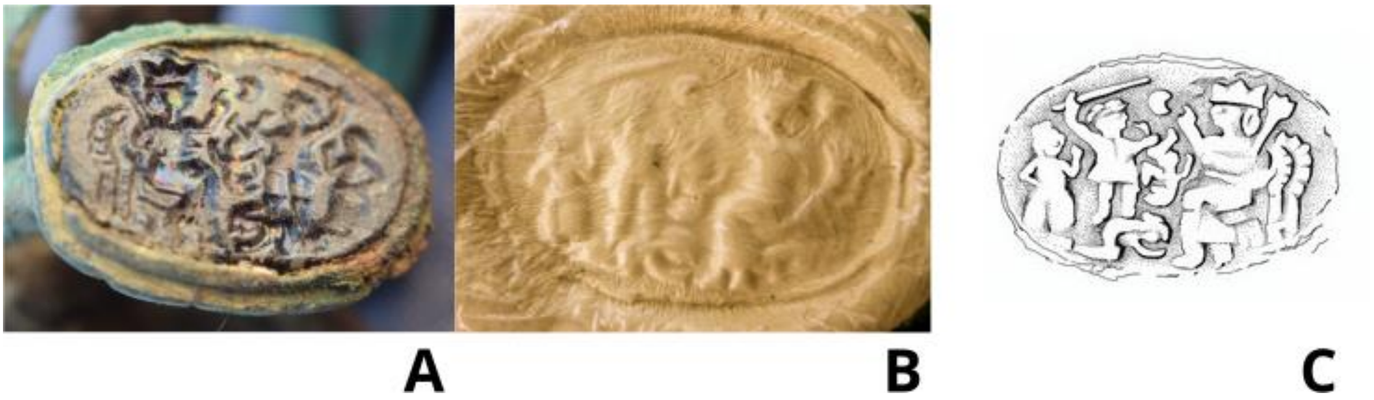

Supplementary Figure 3 – The judgment of Solomon
A: The embossed signet ring
B: The impression of the engraving
C: The outline of the impression
The scene shows (from right to left on the impression – B and the drawing – C) a King on his throne, bearing a crown and a sceptre, a man holding a child upside down in one hand and a sword in the other, a figure knelt at the feet of the king and on the far left, a (female) figure supporting the man's arm.

Supplementary Note 3: kinship test between At Daban 6 and Sytygane Syhé 1

Likelihood Ratios (LR) were obtained with the Familias software, using the genotypes and allelic frequencies presented in Supplementary Data 2. Allelic frequencies were computed on 41 ancient individuals genotyped at 21 autosomal STR loci. There were no allelic exclusions between the genotypes of At Daban 6 and Sytygane Syhé 1. We tested three hypotheses: the pair are a mother and her child, the pair are siblings, the pair are half siblings. All three hypotheses were compared to a null hypothesis: the pair are unrelated.

The LR of the mother/son hypothesis is 3.6×10^5, which corresponds to a probability of 99.99973%. In the same way, the LR for the full-sibling hypothesis is 1.1×10^4 (a probability of 99.99107%) and LR for the half-sibling hypothesis is 3.5×10^3 (a probability of 99.97144%). The mother/son hypothesis is therefore 33 times more likely than the full-sibling hypothesis, 104 times more likely than the half-sibling hypothesis and 36000 times more likely than the unrelated (null) hypothesis.

The individuals buried at Sytygane Syhé 1 and At Daban 6 share a mitochondrial HV-1 haplotype and belong to haplogroup D5a2a (Supplementary Data 2).

Supplementary Note 4: the *arangas,* a suspended coffin

According to ethnographical and historical data, the ancient Yakut buried only a very small fraction of all deceased individuals. Before Christianisation, most corpses were deposited in *arangas*: laid out on platforms or in coffins on trestles above the ground. Most *arangas* have not been preserved but we discovered the remains of two such structures, although they were devoid of skeletal remains (the bones having been dispersed by local fauna over time). The upper panels (Supplementary Figure 4) show a collapsed *arangas* on the Keluye site, near the river Macha in the region of Nyurba. Among the logs we found blue and white beads, that appeared in Yakutia in the early 18th century and disappeared in the middle of that same century. The lower panels show photographs and the reconstruction of a collapsed *arangas* on the site of Buguyekh.

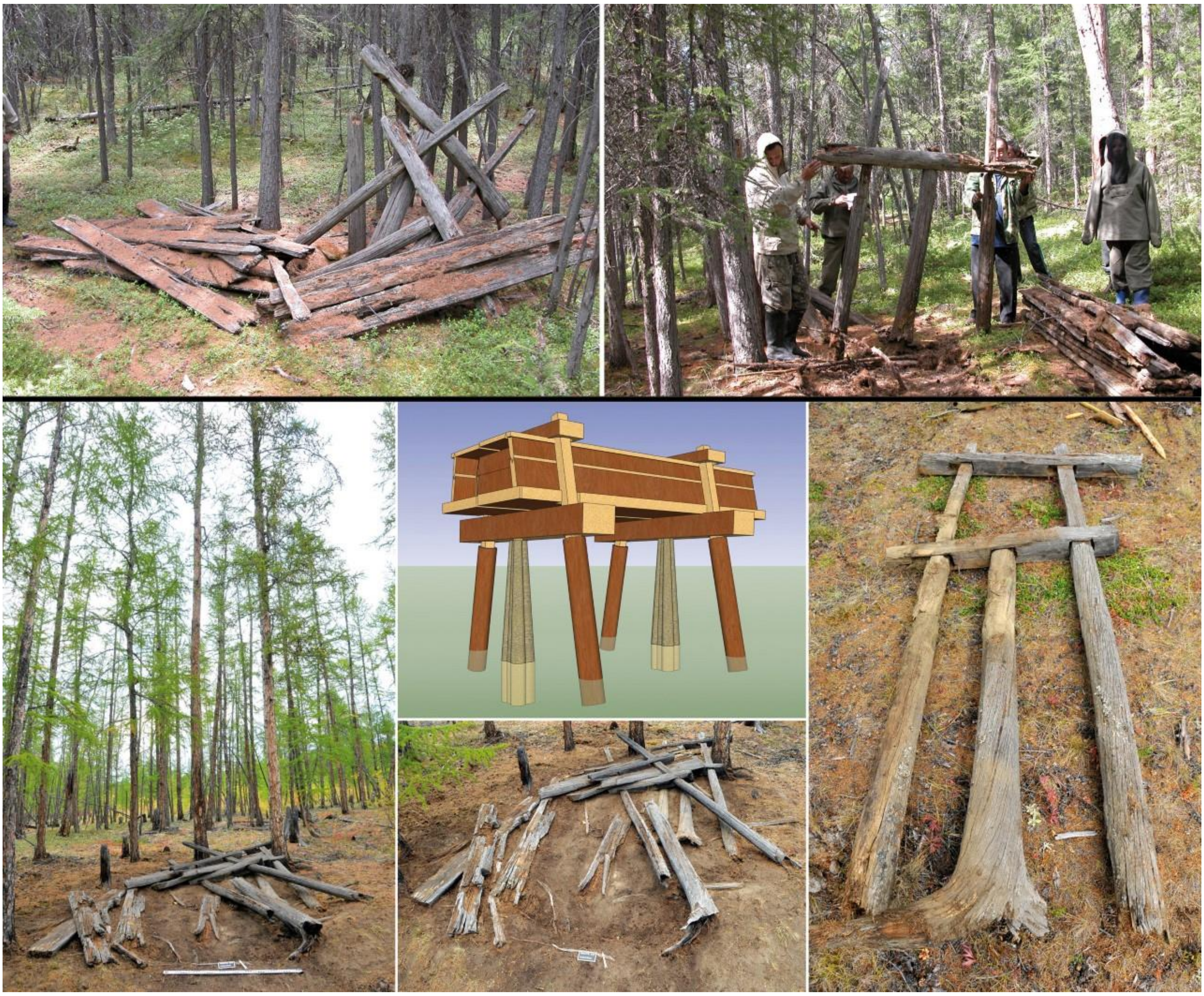

Supplementary Figure 4 - Two collapsed arangas

Supplementary Note 5: archaeological phases (Supplementary Figure 5)

The following is a description of the archaeological material used to distinguish the five recognisable phases of Yakut material history. Supplementary Figure 5 presents a summary of these distinctions, while Supplementary Figures 6 to 9 illustrate the different periods. The graves presented here are the most characteristic of each period.

<u>13th century until 1689/1700</u>

First occurrence of graves in Yakutia in its historical period. We observe great diversity in grave architecture and location, for a small total number of subjects. All subjects found were male and buried with artefacts corresponding to hunters/warriors and, apart from a few small beads, there were no imported goods. Some burials are located on permafrost hills that resemble kurgans. Although the first Russian expeditions reach Yakutia in 1620, no changes in the graves are apparent before the end of the 17th century. In 1689, a trading post with China is established in Nerchinsk. The Yakuts organise illegal imports (contraband) via private caravans (mentions in 1693 and 1698). After the privatisation of pastures, we observe drastic changes in burial locations (cf. infra).

<u>1689/1700 to 1750</u>

This phase is the Yakut "Golden Age". There are large numbers of imported goods in some graves but there is great variability between graves. Both men and women are buried. We observe some differences in grave architecture when it is compared to more ancient or more recent burials.

In 1685, following the victory of one Yakut chieftain associated to the Cossacks over other Yakut chieftains, Yakut society evolves rapidly. Trade intensifies, with the establishment of a trading post in Nerchinsk in 1689. Faced with the growing demand for meat products, cattle breeding develops. Some Yakut chieftains and their wives are now buried near livestock pastures, called *alaas* in Yakut, of which they are the owners. They were privatised and declared transmissible to heirs by two judicial acts, in 1698 and 1701.

According to our data, the period ended around 1750, for several reasons. The first was the decline of the trading post in Nerchinsk, following the establishment or the Kyakhta trading post after the Treaty of Kyakhta in 1728, that took trade away from Yakutia. Then, in 1733, the abolition of slavery diminished the power of Yakut chieftains and great epidemics occurred which destabilised traditional social structure and facilitated Christianisation: smallpox around 1730/1740 and tuberculosis. The disappearance of local taxation institutions in 1753 demonstrates the lack of goods to be taxed: The Golden Age was over.

<u>1750 to 1800</u>

This phase presents a majority of graves of women and children and sees the appearance of the first graveyards. Imported fabrics are more frequent than before but prestigious materials and Chinese beads almost disappear. Offerings disappear from graves and birch bark covers disappear from coffins. These are the first signs of Christianisation, ten to twenty years after the start of the Christianisation campaign of 1737. According to our data, this period ends around 1800.

<u>1800 to 1850/1860</u>

Christian graves appear, usually isolated, that respect Orthodox Christian criteria: instead of grave goods or offerings, a cross. Historically, this is the period of the intensification of Christianisation after the foundation by decree of a school for the Christian education of young Yakuts in 1800 by Emperor Paul I, alongside an increase in the number of popes in the region.

<u>1850/1860 to 1900</u>

Mass Christianisation is decided in 1855 and in five years (by 1860), graveyards have become the norm, although children are sometimes buried in isolated graves, as well as a few adults. In the graves, specific imported items associated to funerary practices are placed: votive candles, psalms written on paper crowns. Graveyards and graves posterior to 1890/1900, some of which used until 1920, are dated by their architecture (tombstones, including some with dates of passing), as well as the presence of shrouds.

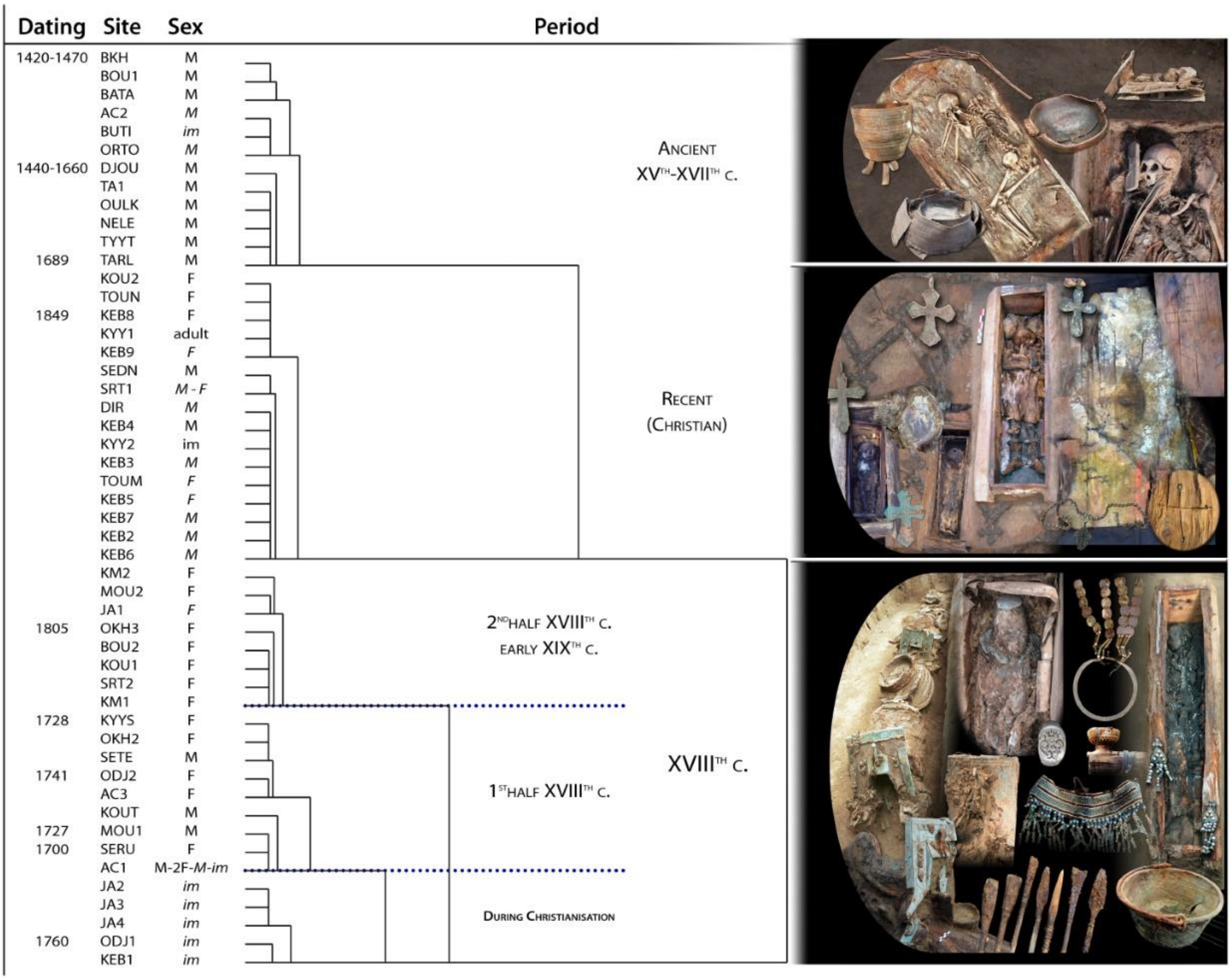

Supplementary Figure 5 – Subjects distributed into periods

50 graves from Central Yakutia with complete archaeological data for 27 categories of items were analysed and grouped in clusters. These clusters can be recognized as chronological phases described in Yakut archaeology and historiography, that are identifiable using items that allow precise dating and the known span of cultural or funerary practices. The addition of absolute dates (column "dating") allows us to refine the divisions and the inclusion of historical data places them in relation with specific events.

M: male; F: female; im: immature

1) The Nelegher grave (before the year 1700)

The Nelegher grave (Supplementary Figure 6), in Central Yakutia, was dated before the 18th century. The man is buried in a coffin composed of logs with a birch bark cover. A palm, a traditional Yakut weapon, was discovered broken in two pieces on top of the coffin. Such breakage was also traditional. The items found were a spoon, left of the subject's head, a shallow bowl, right of his head, and a birch cup against his left leg. He wore a knife in a wooden sheath that was attached to his belt.

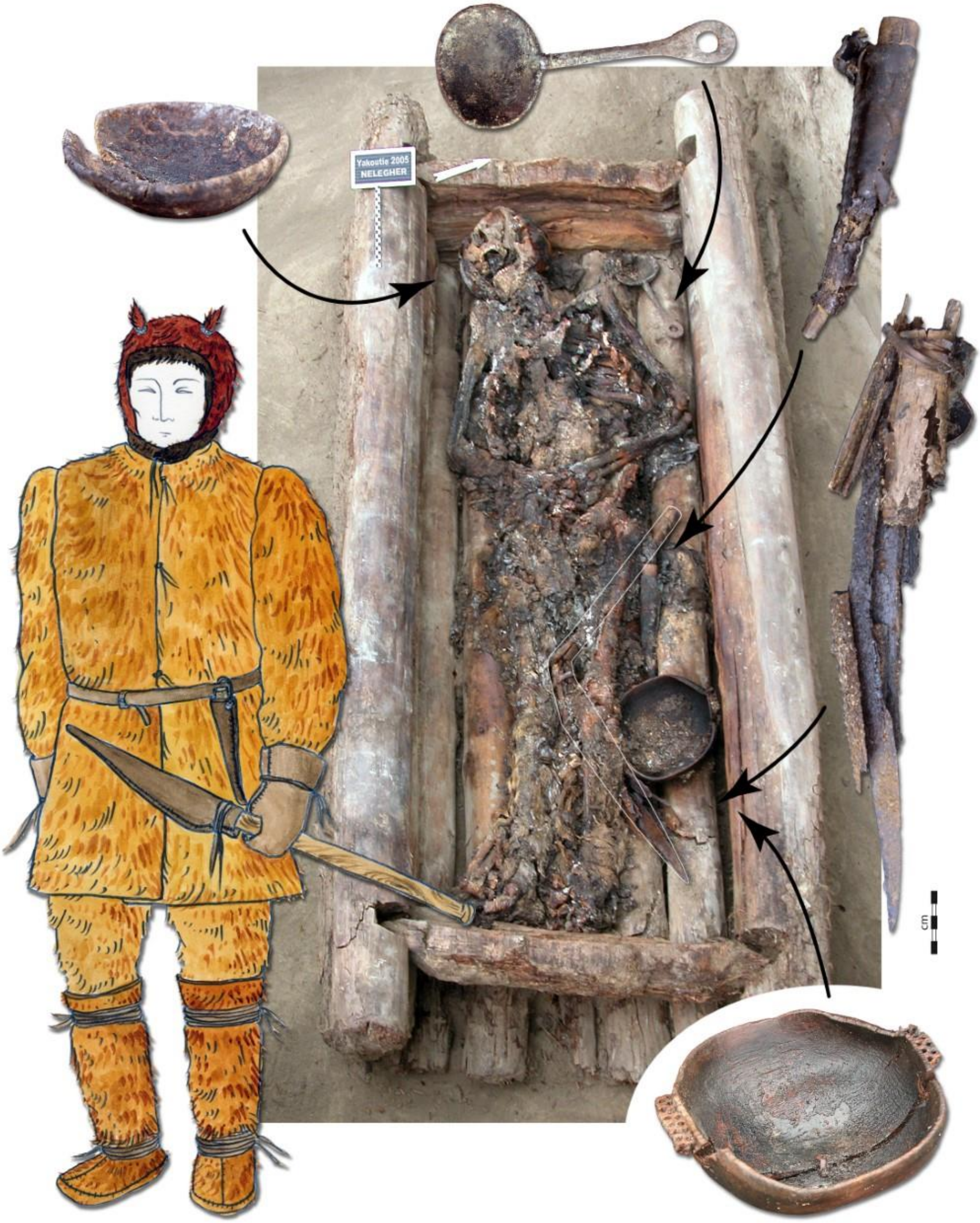

Supplementary Figure 6 – the Nelegher grave

2) The Bakhtakh 3 frozen grave (early 18th century)

Below (Supplementary Figure 7) is the example of the grave of Bakhtakh 3 in the region of Verkhoyansk, dated between 1700 and 1750. In the centre, we propose a reconstitution of the man with his ceremonial vest holding in his hands the *tchoron*, a ritual cup, filled with butter and found in his grave. The three photos of the tomb show different stages of its opening. The relationships between the different elements of the architecture of the grave are illustrated by the computer reconstruction in the upper right corner. The arrows and the *batas* (a Siberian sabre with a long handle – on the bottom of the figure) were placed next to the trunk containing the man. The imported copper cauldron deposited on the subject's legs was a very expensive item reserved to the elite.

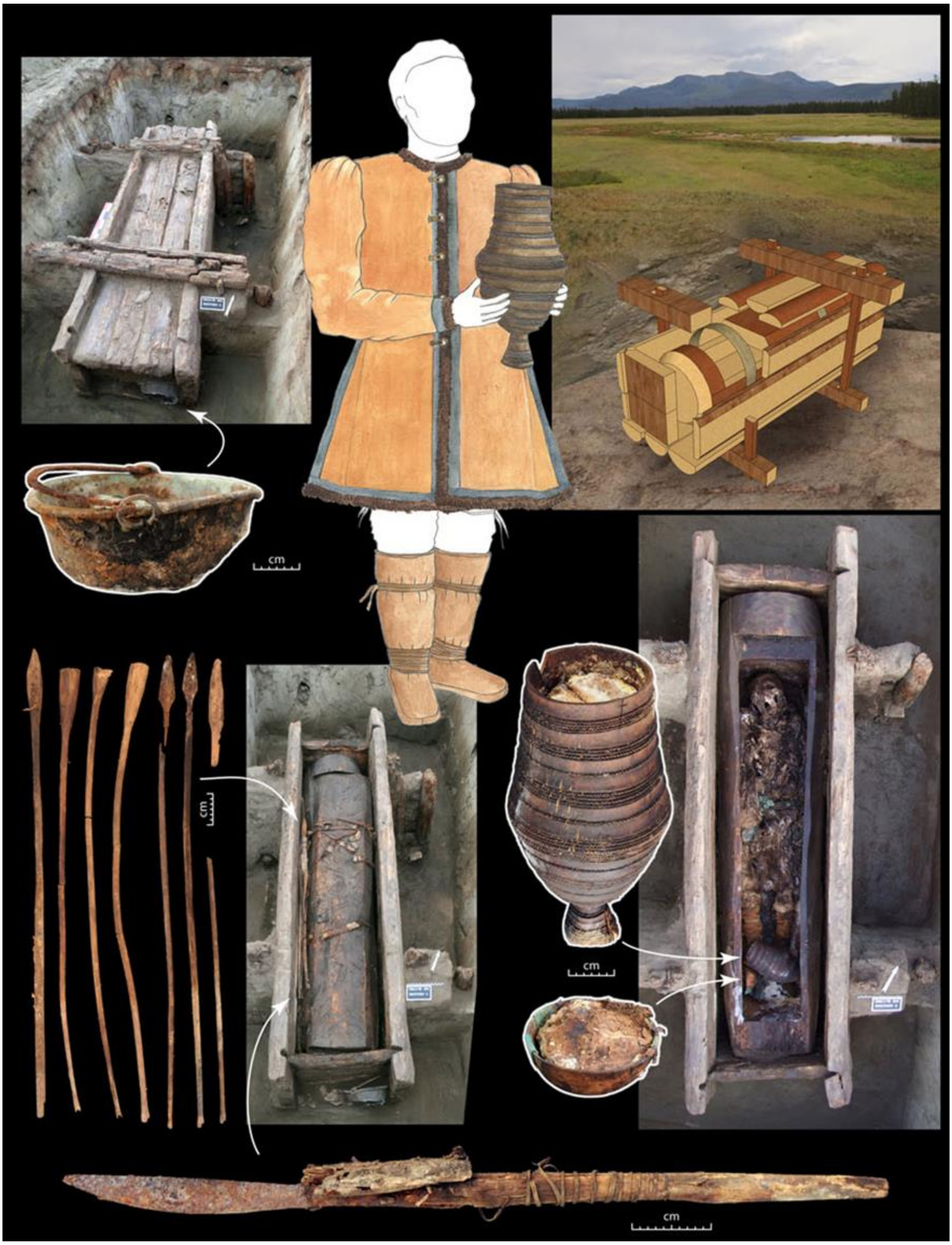

Supplementary Figure 7 – the Bakhtakh 3 frozen grave

3) The Eletchei 1 frozen grave (early 18th century)

Eletchei 1 (Supplementary Figure 8) was a grave in Central Yakutia, dated between 1700 and 1750. A woman was buried there in an imposing double wooden coffin, surmounted by the door of an *ourassa* (summer house), covered in birch bark and very decorated. The space between the coffins (at the feet of the subject) contained a saddle and stirrups, a flywhisk, two travel bags filled with food, as well as wooden and leather containers, and a box containing personal items (a comb and sewing materials). With the subject were deposited a complete set of horse tack, a *knout* (whip) and a pipe. The woman herself is richly clad in a zibeline coat (the back of which shows an eagle motif) and two sumptuous dresses, decorated with several tens of thousands of beads and metal pendants. One of these dresses is made of red wool, a fabric imported from England. Supplementary Figure 8 shows the grave at two stages of opening: first (upper right), the subject is shown wrapped in her shroud and both her coat and dress, then (middle left) she is shown with her second dress (the reconstituted red dress). The items (left to right and top to bottom) are a pipe, a comb, the handle of the flywhisk, a front view of the saddle and the reconstitution of two pots, one of wood and one of birch bark.

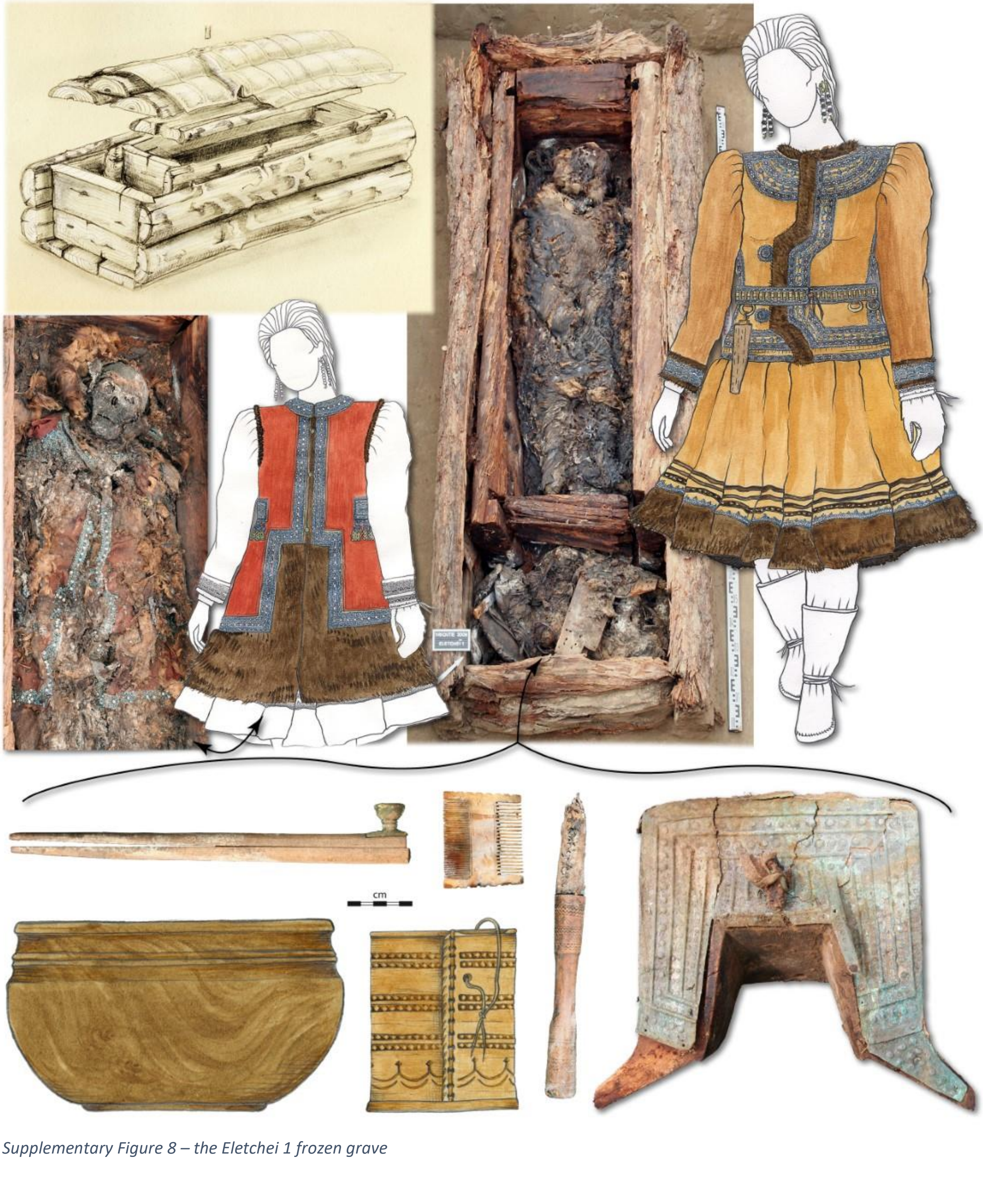

Supplementary Figure 8 – the Eletchei 1 frozen grave

4) The Omouk 1 frozen grave (19th century)

The Omouk 1 grave (Supplementary Figure 9) was found in the region of the Indigirka River and dates from the early 19th century. The grave shows a Christian Orthodox ritual practice (a cross around the neck and votive candles deposited inside the coffin) and European influences, with a Ukrainian headdress. The grave retains characteristics of the late 18th century, such as a superstructure where the ridge of the roof is decorated with horse motifs, a pipe and the corresponding tobacco pouch and fire striker, along with other deposited items. Among these are a teapot or British origin and a cup with a saucer of Russian manufacture, dated from the very beginning of the 19th century. The figure below shows photographs of the structure in situ, the coffin upon opening and a close-up on the subject's chest where votive candles have been deposited. A reconstitution of the coat is included, as well as photos of, in order from left to right: a cross, a pipe, a tobacco pouch, a comb, a teapot, a teacup and saucer.

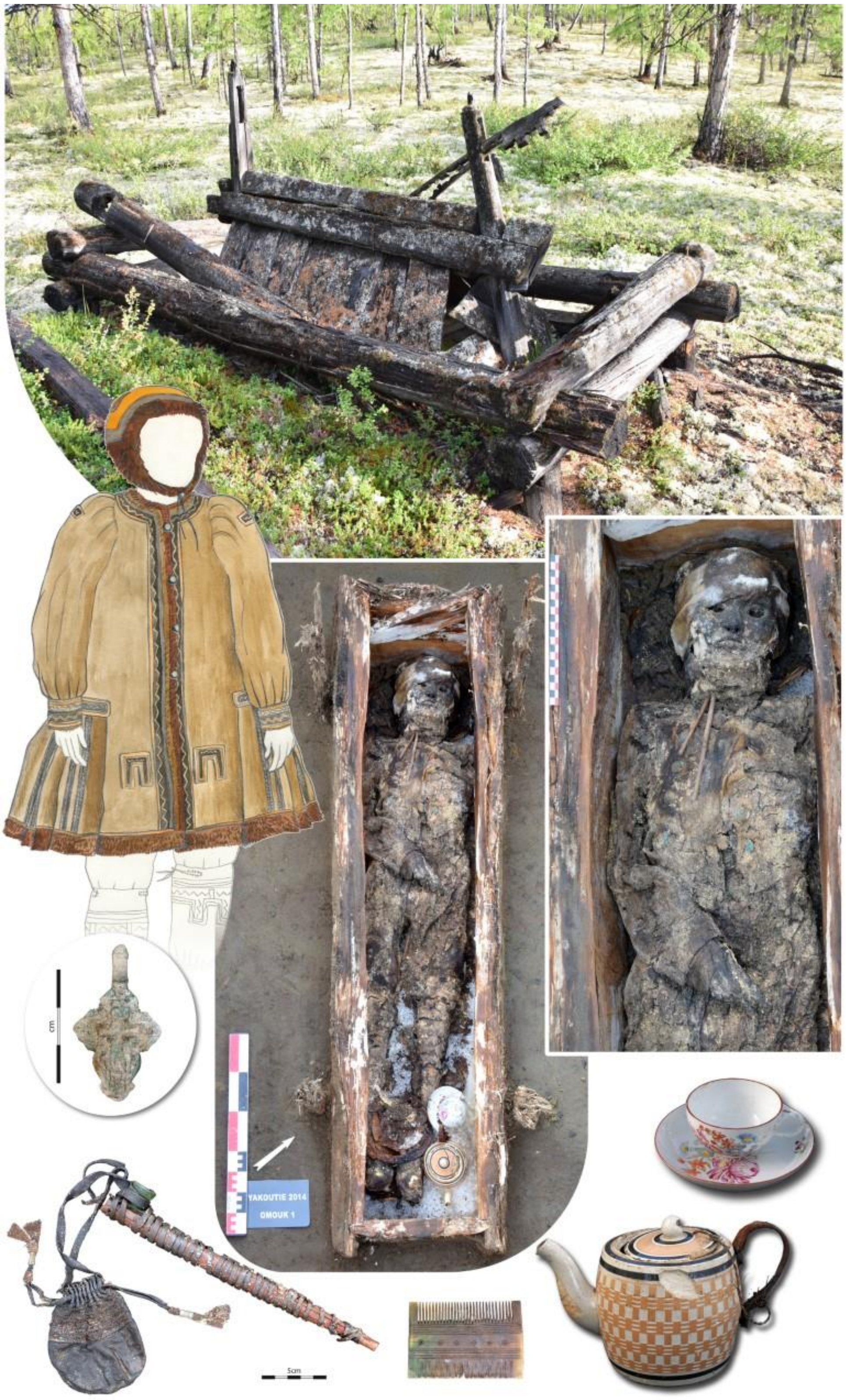

Supplementary Figure 9 – the Omouk 1 frozen grave
