# Supplementary material for: The genetic legacy of legendary and historical Siberian chieftains
Source: Commun Biol. 2020 Oct 16;3:581. doi: 10.1038/s42003-020-01307-3 (PMC7567834; doi:10.1038/s42003-020-01307-3)
Supplement: Supplementary file 3 — Description of Additional Supplementary Files [file 42003_2020_1307_MOESM3_ESM.pdf]

Description of Additional Supplementary Files

File Name: Supplementary Data 1
Description: Y-STR haplotypes

File Name: Supplementary Data 2
Description: Data regarding the mother/son pair

File Name: Supplementary Data 3
Description: Complete mitochondrial haplotypes
