# Supplementary material for: The genetic legacy of legendary and historical Siberian chieftains
Source: Commun Biol. 2020 Oct 16;3:581. doi: 10.1038/s42003-020-01307-3 (PMC7567834; doi:10.1038/s42003-020-01307-3)
Supplement: Supplementary file 7 — Reporting Summary [file 42003_2020_1307_MOESM7_ESM.pdf]

nature research

Corresponding author(s): Vincent Zvenigorosky

Last updated by author(s): Sep 4, 2020

Reporting Summary

Nature Research wishes to improve the reproducibility of the work that we publish. This form provides structure for consistency and transparency in reporting. For further information on Nature Research policies, see our Editorial Policies and the Editorial Policy Checklist.

Statistics

For all statistical analyses, confirm that the following items are present in the figure legend, table legend, main text, or Methods section.

n/a	Confirmed	
☐	☒	The exact sample size (*n*) for each experimental group/condition, given as a discrete number and unit of measurement
☒	☐	A statement on whether measurements were taken from distinct samples or whether the same sample was measured repeatedly
☐	☒	The statistical test(s) used AND whether they are one- or two-sided<br>*Only common tests should be described solely by name; describe more complex techniques in the Methods section.*
☒	☐	A description of all covariates tested
☒	☐	A description of any assumptions or corrections, such as tests of normality and adjustment for multiple comparisons
☒	☐	A full description of the statistical parameters including central tendency (e.g. means) or other basic estimates (e.g. regression coefficient) AND variation (e.g. standard deviation) or associated estimates of uncertainty (e.g. confidence intervals)
☒	☐	For null hypothesis testing, the test statistic (e.g. *F*, *t*, *r*) with confidence intervals, effect sizes, degrees of freedom and *P* value noted<br>*Give P values as exact values whenever suitable.*
☐	☒	For Bayesian analysis, information on the choice of priors and Markov chain Monte Carlo settings
☒	☐	For hierarchical and complex designs, identification of the appropriate level for tests and full reporting of outcomes
☒	☐	Estimates of effect sizes (e.g. Cohen's *d*, Pearson's *r*), indicating how they were calculated

Our web collection on statistics for biologists contains articles on many of the points above.

Software and code

Policy information about availability of computer code

Data collection	No software was used.
Data analysis	Raw genetic data was analyzed using the GeneMapper software (v.4.1). Statistical analyses were performed using the R language (v.3.5 and later versions), the Familias package for R and the BayeSSC program (2005 version). Haplogroups were determined on www.nevgen.org (accessed in december 2019). Network representations were built using the Network software (fluxus-engineering.com).

For manuscripts utilizing custom algorithms or software that are central to the research but not yet described in published literature, software must be made available to editors and reviewers. We strongly encourage code deposition in a community repository (e.g. GitHub). See the Nature Research guidelines for submitting code & software for further information.

Data

Policy information about availability of data

All manuscripts must include a data availability statement. This statement should provide the following information, where applicable:

- Accession codes, unique identifiers, or web links for publicly available datasets
- A list of figures that have associated raw data
- A description of any restrictions on data availability

All data (Y-STR haplotypes, autosomal STR profiles, autosomal STR frequencies) are provided in Supplementary files. Any data that were not used in the present study but relate to the same archaeological sites are available upon request.

Field-specific reporting

Please select the one below that is the best fit for your research. If you are not sure, read the appropriate sections before making your selection.

☒ Life sciences ☐ Behavioural & social sciences ☐ Ecological, evolutionary & environmental sciences

For a reference copy of the document with all sections, see nature.com/documents/nr-reporting-summary-flat.pdf

Life sciences study design

All studies must disclose on these points even when the disclosure is negative.

Sample size	No predetermined sample size was set. Given the difficulties of fieldwork in North-Eastern Siberia, all possible archaeological samples were collected and the collection of modern samples was pursued for the entire duration of the dedicated expeditions.
Data exclusions	No data was excluded.
Replication	Molecular analyses were replicated for each sample. An allele call was only considered valid if it had been consistent and unambiguous over two separate amplifications of two separate extractions. Consensus haplotypes were established only if this criterion was met.
Randomization	No ramdomization. All available samples were included.
Blinding	While no blinding could take place, all analyses originating in different disciplines (anthropology, history, genetics, archaeology, dating) were first conducted separately, in order not to influence one another's approach before confrontation.

Reporting for specific materials, systems and methods

We require information from authors about some types of materials, experimental systems and methods used in many studies. Here, indicate whether each material, system or method listed is relevant to your study. If you are not sure if a list item applies to your research, read the appropriate section before selecting a response.

Materials & experimental systems

n/a	Involved in the study	
☒	☐	Antibodies
☒	☐	Eukaryotic cell lines
☒	☐	Palaeontology and archaeology
☒	☐	Animals and other organisms
☐	☒	Human research participants
☒	☐	Clinical data
☒	☐	Dual use research of concern

Methods

n/a	Involved in the study	
☒	☐	ChIP-seq
☒	☐	Flow cytometry
☒	☐	MRI-based neuroimaging

Human research participants

Policy information about studies involving human research participants

Population characteristics	The participants were volunteer unrelated men living in the Sakha Republic and self-identifying as part of the Yakut ethnic group. There were no age restrictions. In the case of children, their parent or parents provided informed written consent.
Recruitment	Some samples originated in previous studies, with varying strategies. Samples collected specifically for this study were selected in the population of two villages, by establishing complete genealogies and confronting theses genealogies to establish a list of unrelated potential volunteers and identify possible non-Yakut ethnic origins. Participants were invited to discuss all genealogical information that they could remember or had access to.
Ethics oversight	Committee on Biomedical Ethics of the Yakut Scientific Centre of Complex Medical Problems

Note that full information on the approval of the study protocol must also be provided in the manuscript.
